# The Role of Beta2-Microglobulin in Central Nervous System Disease

**DOI:** 10.1007/s10571-024-01481-6

**Published:** 2024-05-14

**Authors:** Zhen-Yuan Liu, Feng Tang, Jin-Zhou Yang, Xi Chen, Ze-Fen Wang, Zhi-Qiang Li

**Affiliations:** 1 Brain Glioma Center & Department of Neurosurgery, Zhongnan Hospital of Wuhan University, Wuhan, Hubei China; 2 Department of Physiology, Wuhan University School of Basic Medical Sciences, Wuhan, Hubei China

**Keywords:** Beta2-microglobulin, Central nervous system diseases, Biomarker

## Abstract

Central nervous system (CNS) disorders represent the leading cause of disability and the second leading cause of death worldwide, and impose a substantial economic burden on society. In recent years, emerging evidence has found that beta2 -microglobulin (B2M), a subunit of major histocompatibility complex class I (MHC-I) molecules, plays a crucial role in the development and progression in certain CNS diseases. On the one hand, intracellular B2M was abnormally upregulated in brain tumors and regulated tumor microenvironments and progression. On the other hand, soluble B2M was also elevated and involved in pathological stages in CNS diseases. Targeted B2M therapy has shown promising outcomes in specific CNS diseases. In this review, we provide a comprehensive summary and discussion of recent advances in understanding the pathological processes involving B2M in CNS diseases (e.g., Alzheimer's disease, aging, stroke, HIV-related dementia, glioma, and primary central nervous system lymphoma).

**Supplementary Information:**

The online version contains supplementary material available at 10.1007/s10571-024-01481-6.

## Introduction

Neurological disorders are a leading cause of disability and the second leading cause of death worldwide (Global, regional, and national burden of neurological disorders during 1990–2015: a systematic analysis for the Global Burden of Disease Study 2015 2017). Central nervous system (CNS) diseases are a range of neurological disorders, including cerebrovascular diseases, brain tumors, neurodegenerative diseases, mental illnesses, and neuroinflammation. Currently, approximately one-sixth of the global population suffers from CNS diseases, and with the growing and ageing population, the economic burden associated with these conditions is expected to increase (Zhou et al. 2021; Global, regional, and national burden of neurological disorders, 1990–2016: a systematic analysis for the Global Burden of Disease Study 2016 2019). Biomedical research has long focused on the pathogenesis of CNS diseases and tried to develop innovative therapies. However, the complex nature of the CNS makes it hard to establish appropriate in vitro and in vivo disease models, which have greatly limited our understanding of the pathology of various CNS diseases (Noch and Khalili 2013). Besides, a variety of potential drugs for the treatment of CNS diseases, especially macromolecular therapeutics, cannot efficiently cross blood–brain-barrier (BBB), which results in poor treatment effectiveness for these diseases (Zhou et al. 2021; Daneman and Prat 2015). In addition, there is still a lack of effective blood biomarkers that can monitor the progression and treatment response of CNS diseases. Therefore, it is meaningful to identify small molecules that can cross the BBB and are involved in CNS diseases, which might have the potential to serve as either serum markers for monitoring the progression of specific CNS diseases or drug target.

β2-Microglobulin (B2M) is a non-glycosylated polypeptide consisting of 119 amino acids with a molecular weight of 11.8 kDa (Ploegh et al. 1981). The secondary structure of B2M is characterized by seven β-strands forming two β-sheets, which are connected by disulfide bonds to form a typical β-sandwich structure. In patients with abnormal renal excretory function, the accumulated B2M folds and aggregates into amyloid fibers deposited in synovium and osteoarticular joints, causing various bone and joint disorders (Goodfellow et al. 1975; Rosano et al. 2005; Eichner and Radford 2011). B2M is synthesized by almost all nucleated cells and associated with the α-chain (heavy chain) of major histocompatibility complex class I (MHC-I) molecules through non-covalent bonds (Shi et al. 2009; Bjorkman et al. 1987). The interaction between B2M and the α-chain is crucial for the transportation of MHC-I molecules from the endoplasmic reticulum to the cell membrane surface (Williams et al. 1989; Sege et al. 1981).

Since B2M is non-covalently linked to the α-chain and not directly attached to the cell membrane, it can detach from the membrane and form free B2M (Schnabl et al. 1990; Glynn et al. 2011). Soluble B2M is widely presented in body fluids, such as cerebrospinal fluid (CSF), urine, and serum (Shi et al. 2009; Peterson et al. 1972). The levels of B2M in serum remain relatively stable, and abnormally elevated B2M levels generally mean the presence of diseases (Huo et al. 2022). Recent studies have shown that serum B2M can serve as a biomarker for multiple diseases, including follicular lymphoma, non-Hodgkin lymphoma, Hodgkin lymphoma, Burkitt lymphoma, diffuse large B-cell lymphoma, and coronary artery disease. Besides, serum B2M are also correlated with tumor grade and prognosis of tumor patients (Federico et al. 2009; Anderson et al. 1983; Dimopoulos et al. 1993; Kim et al. 2021; Chen et al. 2016; You et al. 2017).

Free B2M, as a small molecule, can traverse the blood–brain barrier, thereby impacting the advancement of specific CNS diseases, and is associated with their severity or patient prognosis (Gao et al. 2023; Zhao et al. 2023). In patients with brain injuries, both serum and urine B2M levels were elevated compared to normal controls, correlating with impaired consciousness and cognitive function (Huo et al. 2022). In neonates suffering from hypoxic-ischemic encephalopathy (HIE), levels of B2M in cerebrospinal fluid are significantly increased and correlates with the severity of HIE, which suggests a promising avenue for utilizing B2M as a marker to monitor the extent of inflammation and response to treatment in HIE cases (Carreras et al. 2023). Serum B2M might also serve as one of indicators for distinguishing high-grade gliomas and brain metastases (Li et al. 2020). An increasing number of studies have focused on the roles of B2M in CNS diseases. In this review, we provide a comprehensive summary and discussion of recent advances in understanding the pathological processes involving B2M in CNS diseases.

## Search Strategy and Selection Criteria

In pursuit of relevant literature for their research, two researchers, ZYL and XC, conducted a thorough exploration utilizing PubMed, Scopus, and EMBASE databases. The aim was to identify articles pertinent to their investigation. Any disagreements during the process were addressed through author consultation. The search was limited to English publications. See “Supplementary material” for specific search strategies.

**Fig. 1:**
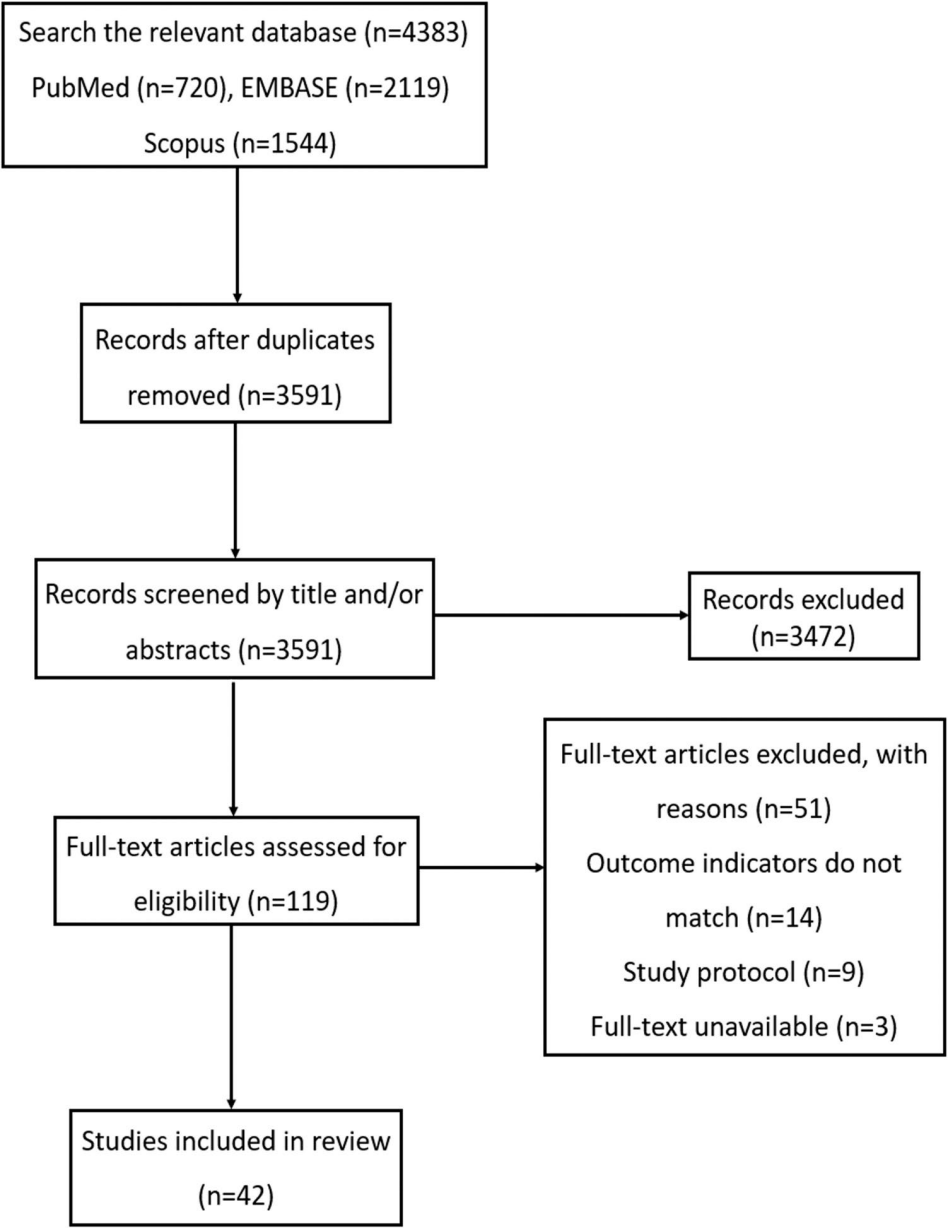
Flowchart illustrating the process of selecting studies that were included in this review

The search process, illustrated in Fig. 1, began with an exhaustive exploration in the specified databases to identify relevant content. Following the removal of duplicate entries, titles and abstracts underwent scrutiny for suitability. Subsequently, full-text articles were assessed against predetermined inclusion and exclusion criteria. A flowchart delineates the rationale behind excluding articles at each stage.

This review focused on key aspects of beta2-microglobulin research within the realm of CNS disorders. Firstly, English-language papers were scrutinized on the topic. Secondly, the association between free B2M and CNS disease research was investigated. Thirdly, observational studies, encompassing both retrospective and prospective approaches, were analyzed. Experimental and animal research methods were applied in the selected studies. Conference papers, research programs, and unpublished works were omitted from consideration. Studies presenting conflicting opinions on potential bias and threats to research quality were also excluded from the analysis. All articles listed in the reference section gained unanimous approval from all authors.

## Basic Biological Functions of B2M

As a subunit of MHC-I molecules, the canonical function of B2M is to maintain the stability of MHC-I and facilitate the presentation of intracellular antigens to cytotoxic T cells (CTLs). During the process of antigen presentation, intracellular proteins or antigens are first degraded into peptides by the proteasome and then transported to the endoplasmic reticulum via the transporter associated with antigen processing (TAP). The peptides subsequently bind to the "shallow groove" of the MHC-I molecule, forming a trimer and is then transported to the cell membrane (Grandea et al. 1995; Adams and Luoma 2013). The peptide-MHC-I complexes present the antigen to cytotoxic T cells or natural killer cells, initiating immune responses (Halle et al. 2017; Orr and Lanier 2010). In the absence of B2M, the heavy chains of MHC-I molecules fail to effectively bind to antigenic peptides, causing accumulation of antigens in the endoplasmic reticulum and impeding their transport to the cell membrane surface (Germain 1994; Ploegh et al. 1979). Consequently, the number of CTLs is reduced, and the maturation of NK is inhibited (Ardeniz et al. 2015; Koller et al. 1990).

## Role of B2M in Specific CNS Diseases

### B2M and Aging

Aging is an inevitable process characterized by a progressive decline in the body's physiological functions and a significant risk factor for various neurodegenerative diseases (Hou et al. 2019). In the CNS, aging leads to a dramatic decline in adult neural stem cells and neurogenesis, accompanied by impairment of cognitive function (Kuhn et al. 1996; Drapeau et al. 2003). Emerging studies demonstrated that peripheral blood-derived factors might influence the aging process. For instance, intravenous injection of plasma from aging mice into young mice resulted in aging-like characteristics, including decreased long-term potentiation (LTP) and hippocampal neurogenesis, coupled with impaired cognitive function (Villeda et al. 2011).

Among these factors, B2M has garnered increasing attention. Research by Smith et al. revealed that levels of B2M in plasma and cerebrospinal fluid were increased with advancing age in healthy individuals, mirroring findings in an aging mouse model. Besides, bound B2M in brain tissues was also higher in old mice compared to that in the younger counterparts (Smith et al. 2015). Dong et al. conducted a comprehensive analysis of B2M and various biochemical indicators in plasma involving 387 healthy individuals, demonstrating a close association between plasma B2M content and aging, with 50 years of age appearing to be a critical point for elevated B2M levels (Dong et al. 2016). In another clinical study involving a smaller sample size of 51 healthy individuals and 41 patients with age-related cognitive impairment, it was evident that the plasma B2M content in patients with cognitive impairment was markedly higher compared to that of the healthy controls (Yang et al. 2017).

Smith and Chen et al. aimed to explore the mechanisms how B2M exerts its roles in promoting aging. The former found that both intravenous and intracranial stereotaxic injection of B2M impaired learning and memory ability of mice by reducing the number of neural stem cells and newborn neurons in the hippocampus (Smith et al. 2015). The latter showed that B2M inhibited hippocampal autophagic flux, thereby impaired cognitive function in rats (Chen et al. 2019). Besides, B2M could also induce cell death and neuroinflammation by activating the TLR4/MyD88/NF-κB signaling pathway in the hippocampal neurons (Zhong et al. 2020). Taken together, these findings emphasize the critical role of B2M as a pro-aging factor that affects cognitive function. Eliminating B2M in plasma represents a potential therapeutic strategy to partially reverse aging-related cognitive impairment.

### B2M and Alzheimer's Disease

Alzheimer's disease (AD) is the most common form of dementia, characterized by the misfolding and aggregation of extracellular β-amyloid (Aβ) and intracellular tubulin-associated tau protein (Long and Holtzman 2019). The accuracy by examining the phosphorylated tau (p-tau), amyloid-β (Aβ_42_) and total tau (T-tau) protein content in CSF has reached 85–90% for diagnosing AD patients (Khan et al. 2020; Blennow and Zetterberg 2018). Notably, the pathophysiological processes of AD initiate several years before clinical diagnosis. Therefore, it is important to find other biomarkers that can be used for early identification and continuous monitoring of AD progression. In an effort to develop future therapeutic interventions for this preclinical stage, the 2011 National Institute on Aging-Alzheimer's Association (NIA-AA) committee proposed a standardized preclinical diagnostic model for AD (Sperling et al. 2011). Huang et al. conducted an extensive analysis of clinical data from preclinical AD patients, unveiling an association between increased plasma B2M levels and the progression of preclinical AD pathological stages. Besides, higher plasma B2M levels correlated with poorer cognitive function (Huang et al. 2023).

In clinical AD patients, CSF B2M levels were higher than that in healthy controls (Davidsson et al. 2002), which was validated by another study (Zhang et al. 2008). Similarly, a large-scale clinical study, which contains two separate cohorts, found that serum B2M was also elevated in AD patients compared to healthy controls (Doecke et al. 2012). Moreover, a retrospective analysis involving 245 subjects (including 45 mild cognitive impairment patients, 100 healthy individuals, and 100 AD patients) revealed that plasma B2M levels was increased in AD patients compared to both normal controls and mild cognitive impairment (MCI) patients (Dominici et al. 2018). In contrast to other serum and CSF biomarkers, result from Bayesian graphical network analysis, a study based on three previously identified collections of multiple AD biomarkers, revealed that B2M was at the central node within the network and exhibited the highest number of connections to other proteins (Rembach et al. 2015). These findings suggest that plasma and CSF B2M concentration might serve as a biomarker aiding in the diagnosis of both the preclinical and clinical AD.

In the study of Huang et al., linear regression analysis of B2M and AD CSF biomarkers indicated a link between elevated plasma B2M levels and Aβ deposition. Mediation analysis further suggested that increased plasma B2M levels may induce Aβ_1-42_ deposition, subsequently leading to cognitive decline (Huang et al. 2023). This study is the first to propose B2M as a key protein implicated in the pathogenesis of AD. Recently, this hypothesis was validated by Zhao et al., who observed that B2M expression was higher in the cerebral cortex of both Alzheimer's disease mice and human patients compared to healthy controls. Further mechanistic research has highlighted that B2M was localized within the core area of amyloid plaques and played crucial roles as a co-aggregation factor of Aβ in triggering AD-related neurodegeneration. Notably, the clearance of B2M in the serum of AD mice effectively improved the pathological characteristics of AD (Zhao et al. 2023), which suggested targeting B2M might as a novel and viable therapeutic strategy for future clinical treatment of AD.

### B2M and Stroke

Stroke, the second leading cause of death worldwide and a major contributor to disability, is caused by numerous risk factors such as heart disease (particularly atrial fibrillation), hypertension, and smoking (Campbell et al. 2019; Delgado et al. 2017). It can be categorized into ischemic and hemorrhagic types, with ischemic stroke resulting from arterial occlusion being the most prevalent, accounting for about 87% of all cases (Tsao et al. 2023). Data from a clinical prospective cohort found that the baseline level of plasma B2M in stroke patients was notably higher compared to the normal control group (Prentice et al. 2010). This result was validated by a subsequent clinical study with an extended follow-up period (Prentice et al. 2013). In women patients with stoke, there was a positive correlation between plasma B2M levels and risk of ischemic stroke (Rist et al. 2017). Interestingly, result from a retrospective study involved 202 cases of acute ischemic stroke (AIS), 41 cases of hemorrhagic stroke, and 253 healthy controls demonstrated that plasma B2M levels in AIS patients were significantly increased compared to those with hemorrhagic stroke and healthy individuals. And B2M exhibited a significant positive correlation with the Essen Stroke Risk Score (ESRS) (Qun et al. 2019).

Notably, regardless of whether ischemic or hemorrhagic stroke, plasma B2M is an independent factor of early prognosis after the onset of stroke, and higher B2M levels is associated with a poorer early prognosis (Hu et al. 2019; Wu et al. 2022). Plasma B2M could also be used to evaluate the extent and degree of acute cerebral infarction (Li and Zhou 2016). Additionally, AIS patients with plasma B2M levels over 2.31 mg/L are more susceptible to disease recurrence, which suggests B2M may act as a potential biomarker for predicting recurrence in these patients (Hu et al. 2022).

Accumulating evidence underscores the significance of inflammation in ischemic stroke (Iadecola et al. 2020; Jayaraj et al. 2019). The NLRP3 inflammasome, a significant mediator, could generate pro-inflammatory factors that orchestrate the inflammatory response and tissue damage in ischemic stroke (Feng et al. 2020). A recent animal study found that following middle cerebral artery occlusion, the levels of B2M, NLRP3, and pro-inflammatory factors (Caspase-1, IL-1β, IL-6, and TNF) in rat brain tissue were significantly increased. Upon B2M knockdown, the levels of NLRP3 and pro-inflammatory factors were markedly decreased. These findings suggest that extracellular free B2M potentially serves as a pro-inflammatory molecule in ischemic stroke by activating the NLRP3 inflammasome and promoting the release of inflammatory factors (Chen et al. 2023).

### B2M and HIV-Related Dementia

In 2015, approximately 37 million people worldwide were infected with the human immunodeficiency virus (HIV), making it one of the leading causes of death globally (Hemelaar et al. 2019; Global, regional, and national age-sex specific all-cause and cause-specific mortality for 240 causes of death, 1990–2013: a systematic analysis for the Global Burden of Disease Study 2013 2015). HIV primarily targets CD4 + T cells, leading to their direct or indirect destruction and hindering regeneration. The progressive loss of CD4 + T cells and consequent immunodeficiency eventually result in acquired immunodeficiency syndrome (AIDS)(Moir et al. 2011; Deeks et al. 2015).

In the early stage of HIV research, serum B2M levels in AIDS patients were found to be significantly higher compared to healthy individuals (Francioli et al. 1982; Bhalla et al. 1983; Grieco et al. 1984). Subsequent studies confirmed that plasma B2M levels continued to increase during the progression of HIV-infected individuals to AIDS. B2M could serve as an independent indicator for predicting the advancement of AIDS, irrespective of the CD4 + T cell count (Zolla-Pazner et al. 1984; Anderson et al. 1990). However, the precise molecular mechanism underlying the elevated plasma B2M levels in AIDS patients remains unidentified. Considering that nearly half of the B2M in plasma originates from lymphocytes, and that lymphocyte activation significantly boosts B2M production (Swanson et al. 1982; Kin et al. 1979), it is reasonable to hypothesize that the heightened B2M levels in AIDS patients result from activated lymphocytes following HIV infection.

Most HIV-infected individuals exhibit cognitive impairments, accompanied by motor dysfunction and behavioral changes. Among these, AIDS dementia complex (ADC) emerges as a severe complication of HIV infection in the CNS, progressively leading to death within a year (Clifford and Ances 2013). HIV does not directly infect neurons but instead affects the chronic inflammatory response of the CNS, contributing to the development of ADC (Clifford 2002), with B2M potentially playing a pivotal role in this process (McArthur et al. 1997). Plasma B2M levels are higher in ADC patients compared to non-ADC patients and serves as a risk factor for ADC progression (Sánchez-Portocarrero et al. 1996; Stern et al. 2001); however, it cannot independently predict neurological outcomes following HIV infection (Childs et al. 1999). In contrast, B2M in the CSF was increased in ADC patients and has been proved to be a valuable indicator of ADC severity (Brew et al. 1992). Interestingly, while non-ADC patients display a strong correlation between serum and CSF B2M levels, ADC patients show increased CSF B2M levels independently of their serum levels (McArthur et al. 1992), suggesting that B2M production in ADC patients originates from the brain. This partly explains why serum B2M cannot predict ADC. Furthermore, abnormally elevated B2M in CSF serves can also reflect the effect of antiretroviral drugs (Brew et al. 1996; Enting et al. 2000).

One hypothesis explaining the association of B2M with ADC suggests that the HIV-envelope glycoprotein binds to unstable HLA-C-specific variant molecules, leading to the dissociation of B2M from HLA-C molecules. The free B2M sustains chronic inflammation in the brain, which, in turn, promotes ADC development (Serena et al. 2017; Zipeto et al. 2018). Though the specific molecular mechanism linking B2M and ADC remains unclear, effective combination antiretroviral treatment (cART) has been successful in inhibiting HIV-1 replication, reducing HIV viral load and neuroinflammation, and significantly reducing the incidence of ADC (Barré-Sinoussi et al. 2013; McArthur 2004).

### B2M and Glioma

Within the realm of oncology, the overexpression of B2M induced the epithelial-mesenchymal transition (EMT) within neoplastic cells, and enhanced the migratory and invasive potential of various cancer types cells including prostate, breast, lung, and renal carcinomas, consequently promoting their metastasis to soft tissues and bone (Josson et al. 2011). In prostate cancer, B2M instigated the cAMP-dependent PKA signaling pathway, thereby facilitating the proliferation and survival of cancer cells within the bone microenvironment (Huang et al. 2006). Employing B2M-targeting antibody promoted prostate cancer cell death through the activation of the caspase-9-dependent pathway (Huang et al. 2008). Similarly, in human renal cell carcinoma (RCC) cells, B2M suppressed cell death by activating the PI3K/Akt, ERK, and JNK signaling pathways (Nomura et al. 2007). These findings highlighted the extensive effects of B2M on the progression of solid tumors.

In addition to above types of tumors, B2M was also abnormally expressed in the glioma, the primary malignant brain tumor with the highest lethality in adults. From the central region of the glioma to its periphery, there is a progressive enhancement in the invasive potential of glioma cells. Conversely, the expression of B2M, along with the levels of MHC-I and class II molecules within these cells, undergoes sequential reduction (Zagzag et al. 2005). Recent studies by our research team and others have found that B2M mRNA and protein levels were significantly elevated in glioma when compared to normal brain tissue (Tang et al. 2021; Zhang et al. 2021). The expression levels of B2M exhibited a positive correlation with the malignancy grade of glioma, and higher B2M expression was related to a poorer prognosis in glioma patients. In addition, using bioinformatics methods, we analyzed multiple publicly available glioma databases and found that B2M might promote glioma progression by modulating the tumor immune microenvironment (Tang et al. 2021). Consistent with our results, recent research has illuminated the pivotal role of B2M in maintaining the properties of glioma stem cells (GSCs). Furthermore, B2M in GSCs could activate the PI3K/AKT/MYC signaling axis to stimulate TGF-β1 secretion, thereby induced AKT pathway activation in macrophages to promote their polarization into M2 types, which, in turn, facilitated glioma progression (Li et al. 2022).

### B2M and Primary Central Nervous System Lymphoma

Primary central nervous system lymphoma (PCNSL) is a rare, highly invasive extranodal non-Hodgkin lymphoma (NHL) that accounts for approximately 4% of intracranial tumors (Villano et al. 2011). Despite its low occurrence, PCNSL is characterized by heightened invasiveness and a propensity for metastasis, resulting in a dismal prognosis (Grommes and DeAngelis 2017). A higher prevalence of MHC-I molecule loss in CNS lymphomas was found compared to nodal lymphomas (Riemersma et al. 2000). And mutations in the *B2M* leading to decreased or absent B2M expression in CNS lymphoma might be a crucial factor, which, in turn, promoted tumor cells to evade immune surveillance (Jordanova et al. 2003).

Previous study has demonstrated that lymphoma patients exhibit a significant elevation in B2M levels in CSF compared to normal controls. Interestingly, levels of serum B2M in patients with CNS lymphoma were also higher than that in non-CNS lymphoma patients (Mavlight et al. 1980; Storti et al. 1986). Furthermore, the levels of B2M in the CSF of CNS lymphoma patients was found to be significantly higher than those with GBM and other brain tumors (Maeyama et al. 2020). Given similarities of the clinical presentation and radiological findings among CNS lymphoma, gliomas and metastatic tumors, it is challenging to distinguish CNS lymphoma from other types of brain tumors solely based on MRI imaging (Nagashima et al. 2018). Therefore, CSF B2M levels hold promise as an adjunctive diagnostic indicator for CNS lymphoma (Inoue et al. 2023). Besides, in both univariate and multivariate Cox analyses, plasma B2M levels in CNS lymphoma patients was also found to be associated with prognosis (Wu et al. 2023). These findings suggest that B2M levels in plasma might serve as an indicator for distinguishing CNS lymphoma from other types of intracranial tumors and non-CNS lymphoma, and predicting patient outcomes in CNS lymphoma.

## Conclusions and Prospects

**Fig. 2:**
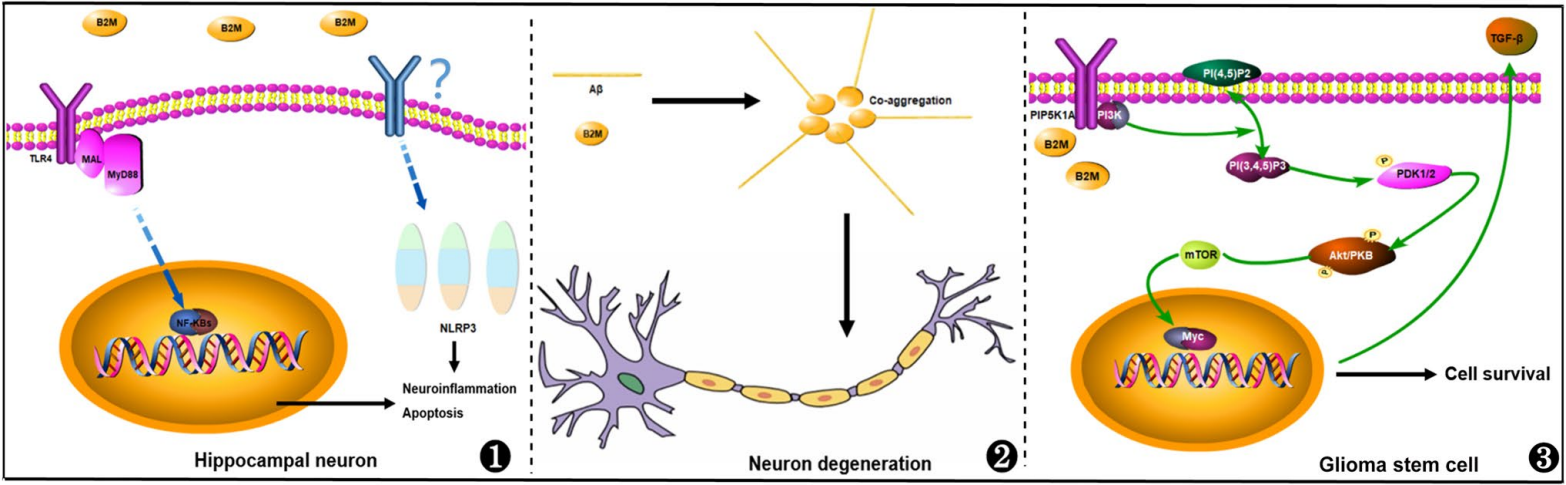
The molecular mechanism of B2M in neurons and glioma stem cells

As a subunit of MHC-I, the immunomodulatory function of B2M has been fully studied, and it is expected to become a potential target for cancer immunotherapy (Wang et al. 2021). Meanwhile, recent research has shown that B2M seemed to be more involved in the initiation and progression of certain CNS diseases in the form of free small molecules. In this review, we summarized and discussed the roles and possible clinical applications of free B2M within specific CNS disorders. Available evidence suggested that levels of B2M in most patients with CNS diseases were elevated and were closely associated with disease progression or severity (Table 1). The possible mechanisms by which B2M played a role might involve signaling pathways that regulated neuronal activity and tumor cells (Fig. 2).

**Table 1:** Summary of expression and possible roles of B2M in CNS diseases

Diseases	B2M levels	Tissues	Models	Effect	Prognosis	References
Aging	Increase	Plasma, CSF, Brain tissue	Human, Mouse	Cognitive impairment	NA	Smith et al. (2015), Dong et al. (2016), and Yang et al. (2017)
Brain injury	Increase	Plasma, Urine	Human	Cognitive impairment	NA	Huo et al. (2022)
Hypoxic-ischemic encephalopathy	Increase	CSF	Human infants	Brain inflammation	NA	Carreras et al. (2023)
Alzheimer's Disease	Increase	Plasma, CSF, Brain tissue	Human, Mouse	Cognitive impairment	NA	Zhao et al. (2023), Huang et al. (2023), Davidsson et al. (2002), Zhang et al. (2008), Doecke et al. (2012), and Dominici et al. (2018)
Acute cerebral infarction	Increase	Plasma, CSF, Brain tissue	Human, Rat	Brain damage and cognitive impairment	NA	Li and Zhou (2016) and Chen et al. (2023)
Stroke	Increase	Plasma	Human	Biomarker	Poor	Prentice et al. (2010), Prentice et al. (2013), Rist et al. (2017), Qun et al. (2019), Hu et al. (2019), and Wu et al. (2022)
AIDS dementia	Increase	Plasma, CSF	Human	Cognitive impairment	NA	Sánchez-Portocarrero et al. (1996), Stern et al. (2001), and Brew et al. (1992)
CNS lymphoma	Increase	CSF	Human	Biomarker	Poor	Mavlight et al. (1980), Storti et al. (1986), and Maeyama et al. (2020)

*B2M* β2-microglobulin, *CNS* central nervous system, *CSF* cerebrospinal fluid

Previous evidence has found that MHC-I molecules, present on the surface of neuronal cells during CNS development and maturation, play a critical in regulating brain development, synaptic plasticity, and social behavior in animals, independently of their typical immune function (Boulanger and Shatz 2004). However, it should be noted that these studies only utilized *B2M*-null mice or *B2M*-knockout neuron models to examine changes in the physiological function of neurons lacking MHC-I molecules (Loconto et al. 2003; Huh et al. 2000; Corriveau et al. 1998; Goddard et al. 2007). Caution is needed in interpreting these findings, as these models not only altered the amount of MHC-I on the surface of neurons, but also reduced free B2M levels in CNS. Therefore, the results observed in these studies might not only be mediated by MHC-I molecules. For instance, Glynn MW et al. revealed that during the initial establishment of connections in the CNS, MHC-I molecules blocked synaptic connections in the cerebral cortex by inhibiting the density and signal strength of glutamate synapses. This effect was not mediated by the intact MHC-I (containing B2M) molecules but by the MHC-I heavy chain (lacking B2M). That is, this inhibitory function was nullified when free B2M was combined with MHC-I heavy chains (Glynn et al. 2011). In addition, using the Down syndrome model, a recent study found that B2M acted as a neuronal NMDA receptor antagonist to impair neuronal synaptic function and cognitive ability in mice independently of the MHC-I molecule (Gao et al. 2023). These results indicated that the free B2M might play a regulating function in CNS development.

Most of the research related to B2M in CNS diseases comes from clinical studies, emphasizing its potential value for clinical translation. Regarding diagnostics, nearly all of the aforementioned studies reported abnormally elevated B2M levels in the CSF or serum of patients with CNS diseases, suggesting that B2M could be used as an indicator to assist in the clinical diagnosis of the diseases. Investigating disease diagnostic models incorporating free B2M and evaluating their sensitivity and specificity represented a valuable direction for future clinical research. Concerning treatment, the findings of B2M in Alzheimer's disease, stroke, ageing, and gliomas highlighted the therapeutic potential of B2M in CNS diseases. In particular, B2M could traverse the blood–brain barrier and preliminary studies confirmed that targeting B2M in the serum partially countered its impact on the CNS. Furthermore, fluctuations in B2M levels within serum and CSF might also serve as indicators of alterations in the condition of specific CNS diseases and responses to treatment interventions.

While significant progress has been made in researching B2M in CNS diseases, several unresolved issues remain for future investigation. For instance, studies of B2M in other neurodegenerative diseases other than Alzheimer's disease were limited. Currently, there was only one published study examining the association between B2M and Parkinson's disease, which reported a notably elevated B2M concentration in the CSF of Parkinson's patients, even surpassing that of Alzheimer's disease patients (Zhang et al. 2008). The involvement of B2M in the progression of Parkinson's disease and other neurodegenerative disorders, as well as its potential utility as a diagnostic adjunct and prognostic indicator, remain unknown.

The impact of B2M on psychiatric disorders is also an intriguing question. There seems to be a shift in the immune response of patients with schizophrenia fromTh1-like cells to Th2-like cells (Schwarz et al. 2001). In vitro experiments have revealed that elevated levels of B2M inhibited dendritic cell differentiation and impeded its antigen-presenting function, thereby impacting Th1-like cellular responses (Xie et al. 2003). Consistent with that, compared to appropriately matched healthy controls, individuals with schizophrenia exhibited higher serum B2M levels (Chittiprol et al. 2009). These findings indicated that elevated B2M levels might contribute to the pathogenesis of schizophrenia. Nevertheless, research on B2M in psychiatric disorders remains scarce and needs further exploration. Even in conditions such as Alzheimer's disease, gliomas, and ageing, while recent research indicates a critical role for B2M in these diseases, a more comprehensive understanding of the mechanisms underlying B2M's function in the development and progression of these conditions is imperative.

In summary, despite the existing challenges, considering B2M as a potential biomarker and therapeutic target for CNS diseases holds considerable appeal, and it merits further extensive research in the future.

## Supplementary Information

Below is the link to the electronic supplementary material.

Supplementary file1 (DOCX 14 kb)

## Data Availability

Data sharing not applicable to this article as no datasets were generated or analyzed during the current study.

## Abbreviations

AD: Alzheimer’s disease
ADC: AIDS dementia complex
B2M: Beta2-microglobulin
BBB: Blood–brain barrier
CNS: Central nervous system
CSF: Cerebrospinal fluid
GSCs: Glioma stem cells
MHC-I: Major histocompatibility complex-I
PCNSL: Primary central nervous system lymphoma
