# Supplementary material for: The Role of Beta2-Microglobulin in Central Nervous System Disease
Source: Cell Mol Neurobiol. 2024 May 14;44:46. doi: 10.1007/s10571-024-01481-6 (PMC11093819; doi:10.1007/s10571-024-01481-6)
Supplement: Supplementary file 1 — Supplementary file1 (DOCX 14 kb) [file 10571_2024_1481_MOESM1_ESM.docx]

Specific search strategies

Use the following title terms and text words to screen the literature independently:

#1 (B2M) OR (Beta2-Microglobulin);

#2 (CNS Disease) OR (CNS Diseases) OR (Central Nervous System Disease) OR (Central Nervous System Diseases) OR (Central Nervous System Disorder) OR (Central Nervous System Disorders);

#3 (Neurodegenerative Disease) OR (Degenerative Diseases, Neurologic) OR (Neurologic Degenerative Disease) OR (Degenerative Neurologic Diseases) OR (Degenerative Neurologic Disease) OR (Neurologic Disease, Degenerative) OR (Neurologic Diseases, Degenerative) OR (Nervous System Degenerative Diseases) OR (Neurodegenerative Disorders) OR (Neurodegenerative Disorder) OR (Neurologic Degenerative Conditions) OR (Degenerative Condition, Neurologic) OR (Degenerative Conditions, Neurologic) OR (Neurologic Degenerative Condition) OR (Neurologic Degenerative Diseases) OR (Degenerative Diseases, Nervous System) OR (Degenerative Neurologic Disorders) OR (Degenerative Neurologic Disorder) OR (Neurologic Disorder, Degenerative) OR (Neurologic Disorders, Degenerative) OR (Degenerative Diseases, Spinal Cord) OR (Degenerative Diseases, Central Nervous System);

#4 (Aging) OR (Senescence);

#5 (Alzheimer's Disease) OR (Alzheimer Dementia) OR (Alzheimer Dementias) OR (Dementia, Alzheimer) OR (Dementia, Senile) OR (Senile Dementia) OR (Dementia, Alzheimer Type) OR (Alzheimer Type Dementia) OR (Alzheimer-Type Dementia (ATD)) OR (Alzheimer Type Dementia (ATD)) OR (Dementia, Alzheimer-Type (ATD)) OR (Alzheimer Type Senile Dementia) OR (Primary Senile Degenerative Dementia) OR (Dementia, Primary Senile Degenerative) OR (Alzheimer Sclerosis) OR (Sclerosis, Alzheimer) OR (Alzheimer Syndrome) OR (Alzheimer's Diseases) OR (Alzheimer Diseases) OR (Alzheimers Diseases) OR (Senile Dementia, Alzheimer Type) OR (Acute Confusional Senile Dementia) OR (Senile Dementia, Acute Confusional) OR (Dementia, Presenile) OR (Presenile Dementia) OR (Alzheimer Disease, Late Onset) OR (Late Onset Alzheimer Disease) OR (Alzheimer's Disease, Focal Onset) OR (Focal Onset Alzheimer's Diseas)e OR (Familial Alzheimer Disease (FAD)) OR (Alzheimer Disease, Familial (FAD)) OR (Familial Alzheimer Diseases (FAD)) OR (Alzheimer Disease, Early Onset) OR (Early Onset Alzheimer Disease) OR (Presenile Alzheimer Dementia);

#6 (Stroke) OR (Strokes) OR (Cerebrovascular Accident) OR (Cerebrovascular Accidents) OR (CVA (Cerebrovascular Accident)) OR (CVAs (Cerebrovascular Accident)) OR (Cerebrovascular Apoplexy) OR (Apoplexy, Cerebrovascular) OR (Vascular Accident, Brain) OR (Brain Vascular Accident) OR (Brain Vascular Accidents) OR (Vascular Accidents, Brain) OR (Cerebrovascular Accidents, Acute) OR (Hemorrhage, Cerebrum) OR (Cerebrum Hemorrhage) OR (Cerebrum Hemorrhages) OR (Hemorrhages, Cerebrum) OR (Cerebral Parenchymal Hemorrhage) OR (Cerebral Parenchymal Hemorrhages) OR (Parenchymal Hemorrhage, Cerebral) OR (Parenchymal Hemorrhages, Cerebral) OR (Intracerebral Hemorrhage) OR (Hemorrhage, Intracerebral) OR (Hemorrhages, Intracerebral) OR (Intracerebral Hemorrhages) OR (Hemorrhage, Cerebral) OR (Cerebral Hemorrhages) OR (Hemorrhages, Cerebral) OR (Brain Hemorrhage, Cerebral) OR (Brain Hemorrhages, Cerebral) OR (Cerebral Brain Hemorrhage) OR (Cerebral Brain Hemorrhages) OR (Hemorrhage, Cerebral Brain) OR (Hemorrhages, Cerebral Brain) OR (Brain Ischemias) OR (Ischemia, Brain) OR (Ischemic Encephalopathy) OR (Encephalopathy, Ischemic) OR (Ischemic Encephalopathies) OR (Cerebral Ischemia) OR (Cerebral Ischemias) OR (Ischemias, Cerebral) OR (Ischemia, Cerebral) OR (Cerebral Infarctions)OR (Infarctions, Cerebral) OR (Infarction, Cerebral) OR (Cerebral Infarct) OR (Cerebral Infarcts) OR (Infarct, Cerebral) OR (Infarcts, Cerebral) OR (Cerebral Infarction, Left Hemisphere) OR (Left Hemisphere, Infarction, Cerebral) OR (Infarction, Left Hemisphere, Cerebral) OR (Left Hemisphere, Cerebral Infarction) OR (Cerebral, Left Hemisphere, Infarction) OR (Infarction, Cerebral, Left Hemisphere) OR (Subcortical Infarction) OR (Infarction, Subcortical) OR (Infarctions, Subcortical) OR (Subcortical Infarctions) OR (Posterior Choroidal Artery Infarction) OR ()Anterior Choroidal Artery Infarction OR (Cerebral Infarction, Right Hemisphere) OR (Right Hemisphere, Cerebral Infarction) OR (Infarction, Right Hemisphere, Cerebral) OR (Right Hemisphere, Infarction, Cerebral) OR (Cerebral, Right Hemisphere, Infarction) OR (Infarction, Cerebral, Right Hemisphere);

#7 (AIDS dementia complex) OR (Complex, AIDS Dementia) OR ()Dementia Complex, AIDS OR (HIV-Associated Cognitive Motor Complex) OR (HIV Associated Cognitive Motor Complex) OR (Dementia Complex, Acquired Immune Deficiency Syndrome) OR (Acquired-Immune Deficiency Syndrome Dementia Complex) OR (AIDS-Related Dementia Complex) OR (AIDS Related Dementia Complex )OR (HIV Dementia) OR (Dementia, HIV) OR (Dementias, HIV) OR (HIV Dementias) OR (Dementia Complex, AIDS-Related) OR (Complex, AIDS-Related Dementia) OR (Dementia Complex, AIDS Related) OR (HIV-1-Associated Cognitive Motor Complex) OR (HIV 1 Associated Cognitive Motor Complex) OR (HIV-1 Cognitive and Motor Complex) OR (HIV 1 Cognitive and Motor Complex) OR (HIV Encephalopathy) OR (Encephalopathies, HIV) OR (HIV Encephalopathies) OR (Encephalopathy, AIDS) OR (Encephalopathy, HIV) OR (AIDS Encephalopathy) OR (AIDS Encephalopathies) OR (Encephalopathies, AIDS);

#8 (Glioma) OR (Gliomas) OR (Glial Cell Tumors) OR (Glial Cell Tumor) OR (Tumor, Glial Cell) OR (Tumors, Glial Cell) OR (Astrocytomas) OR (Astroglioma) OR (Astrogliomas) OR (Oligodendrogliomas) OR (Oligodendroglioma) OR (Glioblastoma) OR (Glioblastomas)；

#9 (Primary Central Nervous System Lymphoma);

#10 #2 OR #3 OR #4 OR #5 OR #6 OR #7 OR #8 OR #9

#11 #1 AND #10
